# Evaluation of the inverse electron demand Diels-Alder reaction in rats using a scandium-44-labelled tetrazine for pretargeted PET imaging

**DOI:** 10.1186/s13550-019-0520-y

**Published:** 2019-05-28

**Authors:** Patricia E. Edem, Jean-Philippe Sinnes, Stefanie Pektor, Nicole Bausbacher, Raffaella Rossin, Abdolreza Yazdani, Matthias Miederer, Andreas Kjær, John F. Valliant, Marc S. Robillard, Frank Rösch, Matthias M. Herth

**Affiliations:** 1 Department of Clinical Physiology, Nuclear Medicine & PET, Rigshospitalet, Blegdamsvej 9, 2100 Copenhagen, Denmark; 2 Cluster for Molecular Imaging, Department of Biomedical Sciences, University of Copenhagen, Blegdamsvej 3, 2200 Copenhagen, Denmark; 3 Department of Drug Design and Pharmacology, University of Copenhagen, Jagtvej 162, 2100 Copenhagen, Denmark; 4 Johannes Gutenberg-Universität Mainz, Saarstraße 21, 55122 Mainz, Germany; 5 University Medical Center Mainz, Langenbeckstr. 1, 55131 Mainz, Germany; 6 McMaster University, 1280 Main St. W, Hamilton, ON L8S 4L8 Canada; 7 Tagworks Pharmaceuticals, Geert Grooteplein Zuid 10, 6525 GA Nijmegen, The Netherlands; 8 Pharmaceutical Chemistry and Radiopharmacy Department, School of Pharmacy, Shahid Beheshti University of Medical Sciences, PO Box 14155-6153, Tehran, Iran

**Keywords:** Scandium-44 (^44^Sc), Positron emission tomography (PET), Tetrazine, *Trans*-cyclooctene (TCO), Pretargeted imaging, Inverse electron demand Diels-Alder (IEDDA), Bisphosphonates, Alendronic acid

## Abstract

**Background:**

Pretargeted imaging allows the use of short-lived radionuclides when imaging the accumulation of slow clearing targeting agents such as antibodies. The biotin-(strept)avidin and the bispecific antibody-hapten interactions have been applied in clinical pretargeting studies; unfortunately, these systems led to immunogenic responses in patients. The inverse electron demand Diels-Alder (IEDDA) reaction between a radiolabelled tetrazine (Tz) and a *trans*-cyclooctene (TCO)-functionalized targeting vector is a promising alternative for clinical pretargeted imaging due to its fast reaction kinetics. This strategy was first applied in nuclear medicine using an ^111^In-labelled Tz to image TCO-functionalized antibodies in tumour-bearing mice. Since then, the IEDDA has been used extensively in pretargeted nuclear imaging and radiotherapy; however, these studies have only been performed in mice. Herein, we report the ^44^Sc labelling of a Tz and evaluate it in pretargeted imaging in Wistar rats.

**Results:**

^44^Sc was obtained from an in house ^44^Ti/^44^Sc generator. A 1,4,7,10-tetraazacyclododecane-1,4,7,10-tetraacetic acid (DOTA)-functionalized tetrazine was radiolabelled with ^44^Sc resulting in radiochemical yields of 85–95%, a radiochemical purity > 99% at an apparent molar activity of 1 GBq/mmol. The ^44^Sc-labelled Tz maintained stability in solution for up to 24 h. A TCO-functionalized bisphosphonate, which accumulates in skeletal tissue, was used as a targeting vector to evaluate the ^44^Sc-labelled Tz. Biodistribution data of the ^44^Sc-labelled Tz showed specific uptake (0.9 ± 0.3% ID/g) in the bones (humerus and femur) of rats pre-treated with the TCO-functionalized bisphosphonate. This uptake was not present in rats not receiving pre-treatment (< 0.03% ID/g).

**Conclusions:**

We have prepared a ^44^Sc-labelled Tz and used it in pretargeted PET imaging with rats treated with TCO-functionalized bisphosponates. This allowed for the evaluation of the IEDDA reaction in animals larger than a typical mouse. Non-target accumulation was low, and there was a 30-fold higher bone uptake in the pre-treated rats compared to the non-treated controls. Given its convenient half-life and the ability to perform positron emission tomography with a previously studied DOTA-functionalized Tz, scandium-44 (*t*_1/2_ = 3.97 h) proved to be a suitable radioisotope for this study.

**Electronic supplementary material:**

The online version of this article (10.1186/s13550-019-0520-y) contains supplementary material, which is available to authorized users.

## Background

Personalized medicine (precision medicine) is a practice where the patient’s individual biological/molecular characteristics are used to determine a course of treatment [1]. Although this concept can be traced back to Hippocrates [1], advancements in chemical biology, molecular biology, systems biology, and genomics have inspired resurgence in its practice. Nuclear medicine techniques such as single photon computed tomography (SPECT) and positron emission tomography (PET) play an important role through the use of companion diagnostics in innovative theranostic approaches [2]. Companion diagnostics are agents that can be used to identify a biomarker indicative of a particular disease phenotype. From this information, predictions regarding how a patient may respond to a particular course of treatment can be derived [3]. Unlike other diagnostic tests, radiotracers can be used repeatedly and reflect a particular biologic property throughout the whole body giving a complete view of the disease. This is particularly useful in the case of systemically applied targeted radiotherapy. In this instance, the companion diagnostics (or theranostic) agent is used as a surrogate for the radiotherapeutic. For example, it can be used to evaluate pretherapeutic dosimetry, biodistribution, and maximal tolerated dose prior to administering the radiotherapeutic.

The theranostic approach has been an important factor in the recent developments in radioimmunotherapy mainly due to pretherapeutic dosimetry and measuring treatment response [4]. One of the main drawbacks in radioimmunotherapy though is the sub-optimal pharmacokinetic properties of the antibodies. Due to their long circulation time and slow target accumulation, the radiation burden to the patient can be quite high. This necessitates the use of long-lived radioisotopes further increasing the radiation dose. This can be mitigated with a pretargeting strategy. With this method, there is a temporal separation of the targeting step from the delivery of the radiation at the target site. The process involves a primary agent and a secondary agent, each functionalized with tags that specifically bind to each other in a biological medium. This method can be applied using bioorthogonal chemical reactions such as the inverse electron demand Diels-Alder (IEDDA) reaction, namely the *trans*-cyclooctene (TCO)-tetrazine (Tz) ligation. The first application of this method in nuclear medicine involved a tetrazine labelled with indium-111 via the 1,4,7,10-tetraazacyclododecane-1,4,7,10-tetraacetic acid (DOTA) chelator (**1**) [5]. Following this success, a number of Tz have been developed for diagnostic purposes [6–9], but also a few DOTA-Tz have been labelled with therapeutic isotopes such as lutetium-177 and lead-212 with the hope of providing pretargeted radioimmunotherapy [10–12].

Although any modality can be used to predict the efficacy of a radiotherapeutic, PET is most advantageous due to the level of quantification aiding in more accurate dosimetry measurements. Traditionally, a theranostic in nuclear medicine is an agent consisting of a dual-purpose radionuclide that can be used in both diagnostic and therapeutic applications. However, often times, a radionuclide pair, consisting of either isotopes of the same element or two chemical congeners, can be used as well [13].

For example, scandium-44 is an attractive PET radionuclide for this purpose due to its half-life and emission properties. The half-life for scandium-44 (3.97 h) is long enough for production and transport, yet short enough that radiation doses are minimized. Scandium-44 has a high positron branching ratio (*β*^+^_branch_ = 94.27%) and a suitable positron energy (*E*_*β*,max_ = 1.474) resulting in high image quality for ^44^Sc-labelled radiopharmaceuticals [14, 15]. Furthermore, scandium and lutetium have similar coordination chemistry and the stability of Sc-DOTA complexes is higher than both ^177^Lu-DOTA and the often used ^68^Ga-DOTA [16, 17] [14, 18]. Scandium-44 can be produced from a cyclotron as well as a ^44^Ti/^44^Sc generator. Following these innovations, there has been increased interest in using scandium-44 as a PET alternative to gallium-68 for clinical and preclinical applications [16–19].

**Fig. 1:**
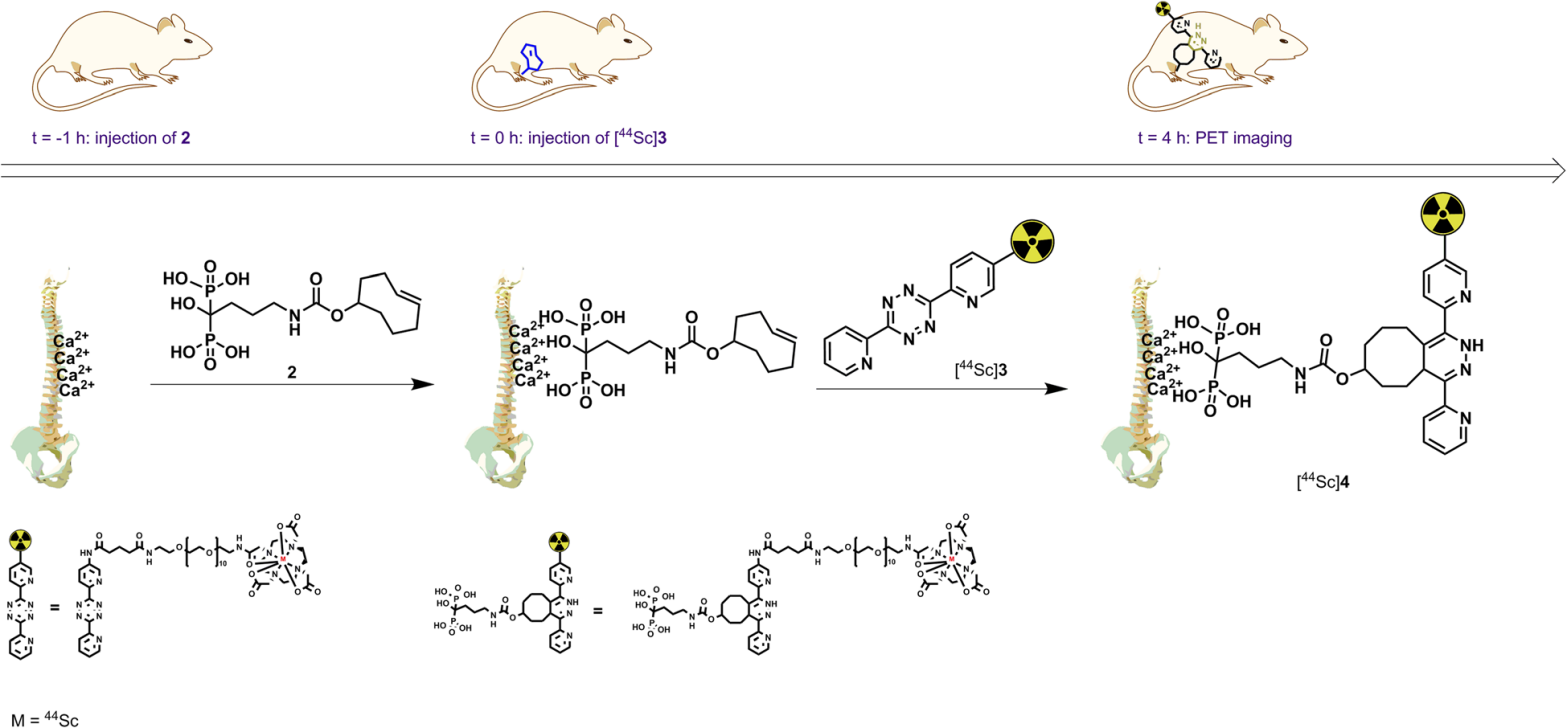
Schematic of the bone-pretargeting strategy. Aln-TCO (**2**) is injected into healthy rats via the tail vein. After 1 h, the radiolabelled tetrazine is injected. PET imaging occurs 4 h after injecting the radiolabelled tracer

The aim of the current study was to develop a ^44^Sc-labelled variant of **1** and evaluate its use for pretargeted PET imaging. The primary agent was a TCO-functionalized variant of the bisphosphonate, alendronic acid (Aln-TCO, **2**) (Fig. 1). Alendronic acid is used in the clinic to treat osteoporosis, and it accumulates in areas with high bone turnover [20]. TCO-modified alendronic acid has been used to screen radiolabelled tetrazines for in vivo pretargeting in healthy mice [21–23] [20, 24, 25]. The possibility to screen in healthy animals is advantageous for applications in higher order species because a disease model is not needed. Furthermore, TCO-modified alendronic acid accumulates at their target sites quickly and exhibits fast clearance from non-targeted tissues. Consequently, clearing agents or lengthy pretargeting lag times are not required as in the case with TCO-functionalized antibodies [21]. One of the challenges in using bioorthogonal reactions for pretargeted imaging is that the reaction kinetics are concentration dependent. This can alter the effectiveness of the reaction when moving from smaller living species to larger ones. For example, early studies applying the Staudinger ligation in vivo were successful in cells, yet the system failed once it was applied to mice models [26–28]. To date, pretargeted imaging using the TCO-Tz ligation has only been reported in mice (ca. 20 g). Various radiolabelled bisphosphonates have been evaluated in healthy Wistar rats as potential agents to image bone metastasis leading to clinical evaluation in patients with skeletal metastases [22, 23, 29, 30]. Therefore, the current pretargeted imaging studies were performed using the larger Wistar rats (ca. 150+ g).

## Methods

### General

All reagents and solvents were purchased from Sigma-Aldrich, unless otherwise stated, and used without further purification. Aln-TCO (**2**) [24] and the DOTA-functionalized tetrazine (**1**) were prepared as previously described [5]. [^44^Sc]ScCl_3_ (120–130 MBq) was obtained from an in house ^44^Ti/^44^Sc generator in 0.25 M sodium acetate pH 4 (3 mL) as described previously [31]. TraceSELECT water (Honeywell) was used for the radiolabelling reactions. Analytical radioactive thin-layer chromatography (radio-TLC) was performed using silica gel plates or C18 plates (Merk) with fluorescent indicator UV254 and visualized using a RITA TLC imager (Elysia Raytest). Analytical high-performance liquid chromatography (HPLC) was performed using a Sykam S 1100 solvent delivery system, S 8110 system equipped with a Dionex UVD 170 U (254 nm) absorbance detector and a Raytest NaI scintillation counter (Gabi) radioactivity detector. Compounds were eluted using a Chromalith Performance RP-18 endcapped 100-4.6 HPLC column using the following elution conditions: solvent A = water with 0.1% trifluoroacetic acid (TFA), solvent B = acetonitrile with 0.1% TFA; gradient 5% B to 95% B, 0–10 min. The flow rate was set at 2.5 mL/min. UV monitoring occurred at 254 nm.

### ^44^Sc labelling

To a vial containing [^44^Sc]ScCl_3_ in 0.25 M NH_4_OAC pH 4 (3 mL), and absolute EtOH (100 μL), **1** (42 μL, 66 nmol) in water was added and heated at 95 °C for 10 min. The reaction was cooled to room temperature (RT), and water (TraceSELECT) was added. The solution was then passed through a preconditioned C-18 SPE cartridge. The cartridge was washed with water (10 mL), and the product ([^44^Sc]**3**) was eluted with 400 μL EtOH. Prior to in vivo evaluation, the eluate was heated until the EtOH content was reduced to < 50 μL and saline (2.4 mL) was added: radiochemical yield (RCY), non-decay corrected (ndc) 85–95%; radiochemical purity (RCP) > 99%; apparent molar activity (*A*_m_) end of synthesis (EOS) 1 GBq/μmol; HPLC *R*_*t*_ = 5.6 min radio-TLC (0.1 M citrate buffer, pH = 4) ([^44^Sc]**3**) *R*_f_ = 0; (free ^44^Sc) *R*_f_ = 0.7–0.8. An aliquot was added to **2** (10 μg, 0.02 μmol) in saline (10 μL). After 5 min, samples were analysed using RP radio-TLC, RP radio-TLC (50% MeCN/H_2_O) *R*_f_ ([^44^Sc]**3**) = 0.9; *R*_f_ ([^44^Sc]**4**) = 0.0.

### Solution stability of [^44^Sc]**3**

Solutions of [^44^Sc]**3** were prepared in saline (*n* = 3) and HSA (*n* = 3) and incubated at 37 °C. Aliquots (*n* = 3) were taken at 0.5, 1, 2, 4, and 24 h and analysed using RP radio-TLC.

### Animal studies

All animal experiments were approved by the ethical committee of the state of Rhineland Palatinate (according to §8 Abs. 1 Tierschutzgesetz, Landesuntersuchungsamt) and performed in accordance with relevant federal laws and institutional guidelines. Male Wistar rats (age 5–6 weeks, mean weight 158 g) were obtained from Janvier and housed in the central animal facility of the Johannes-Gutenberg-University Medical Center, Mainz, Germany, under specific pathogen-free conditions according to current federal, state, and institutional guidelines with free access to water and food. PET imaging was conducted using a Focus 120 (Siemens) scanner.

### Proof-of-concept PET with [^44^Sc]**3**

Rats (168–196 g) were positioned head first supine and were anaesthetized with 2–2.5% isoflurane vaporized in 100% O_2_. Compound **2** (3 mg/kg) in saline (168 μL) (*n* = 1) or saline (196 μL) (*n* = 1) was administered via a peripheral venous catheter inserted into a tail vein. After 1 h, [^44^Sc]**3** (12 MBq, 13–15 μg, 11–12 nmol) in saline was administered and a 60-min dynamic PET scan was started simultaneously. After the emission scan, a 30-min ^57^Co transmission scan was performed. List mode data was rebinned into framed sinograms and subsequently reconstructed using Osem 2D (ramp filter, cut-off = 0.5) into 95 slices of 0.80 mm thickness (pixel size 0.87 × 0.87 mm^2^) and a matrix of 128 × 128 pixels. Corrections were applied for dead time, randoms, attenuation, and scatter. Regions of interest were drawn in three different areas of the left and right shoulders, and the muscle as background. Time-activity curves (TACs) were obtained using image-derived uptake values, calculated as percent injected dose per millilitre (% ID/mL).

### Ex vivo biodistribution with [^44^Sc]**3**

In a similar fashion, rats (165–196 g) were positioned head first supine and were anaesthetized with 2–2.5% isoflurane vaporized in 100% O_2_. Compound **1** (3 mg/kg) in saline (165–186 μL) (*n* = 4) or saline (169–196 μL) (*n* = 2) was administered via a peripheral venous catheter inserted into a tail vein. After 1 h, [^44^Sc]**3** (9–12 MBq, 12–20 μg, 9–15 nmol) in saline was administered. Rats were sacrificed 4 h p.i. by an overdose of isoflurane, and various tissues and organs were harvested and weighed. Sample radioactivity was measured using a gamma counter (Wizard^2^ 2470, PerkinElmer), and the % ID/g values were calculated. Biodistribution data was analysed and plotted using GraphPad Prism (version 7).

## Results

### Radiolabelling and in vitro stability

**Scheme 1:**
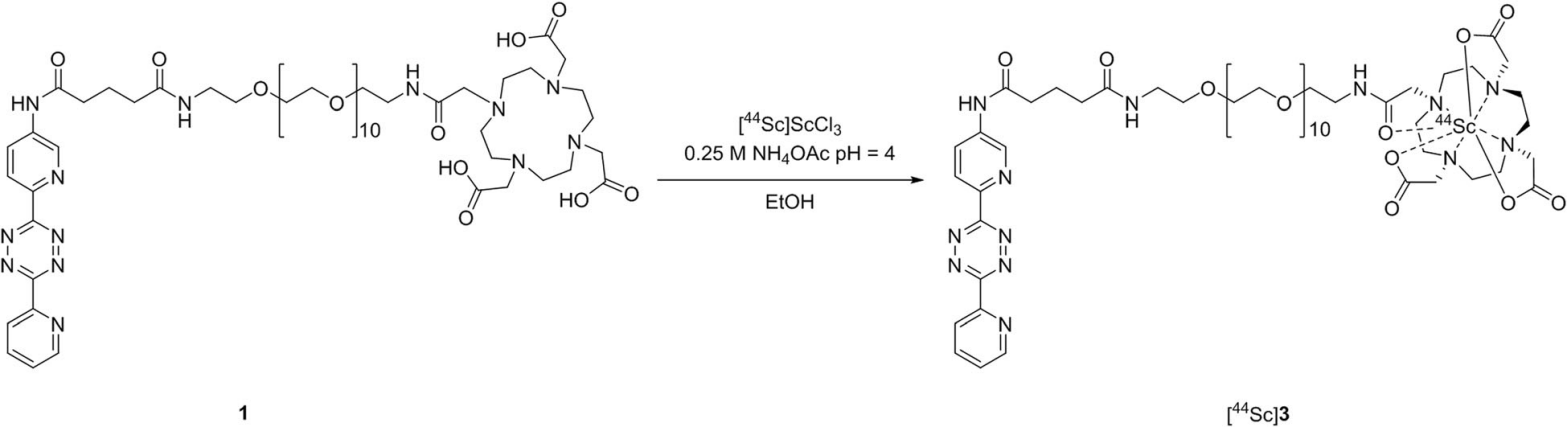
Radiosynthesis of [^44^Sc]**3**. The DOTA-functionalized Tz (**1**) was added to a solution of ^44^ScCl_3_ in ammonium acetate (pH 4) and ethanol. The solution was heated to 95 °C for 10 min. After cooling to room temperature, the solution was loaded on to an SPE column, flushed with water, and the pure product eluted with ethanol. [^44^Sc]**3** was obtained in 85–95% RCY and > 99% RCP.

A brief optimization of the RCY was performed for [^44^Sc]**3**. Initially, the radiolabelling (Scheme 1) was performed by heating a solution of **1** (39 mmol) and [^44^Sc]ScCl_3_ at 95 °C for 20 min, followed by cooling to room temperature and SPE purification. This resulted in RCYs of only 25–28% (ndc). The reaction was analysed at 5, 10, and 20 min reaction times, and it was observed that longer reaction time resulted in the formation of unidentifed side products, present as additional peaks in HPLC chromatograms. A reaction time of 10 min was then chosen to reduce the side products and increased the RCY. The ligand concentration was also increased to 66 nmol to provide a suitable TCO:Tz ratio for in vivo analysis. With these conditions in place, the radiosynthesis of [^44^Sc]**3** was accomplished in high RCYs (85–95% ndc) and RCPs (> 99%), with an apparent *A*_m_ of 1 MBq/nmol (EOS). Compound [^44^Sc]**3** was stable for at least 24 h at 37 °C in saline and HSA (Additional file 1: Figure S2B).

### Proof-of-concept evaluation with PET

**Fig. 2:**
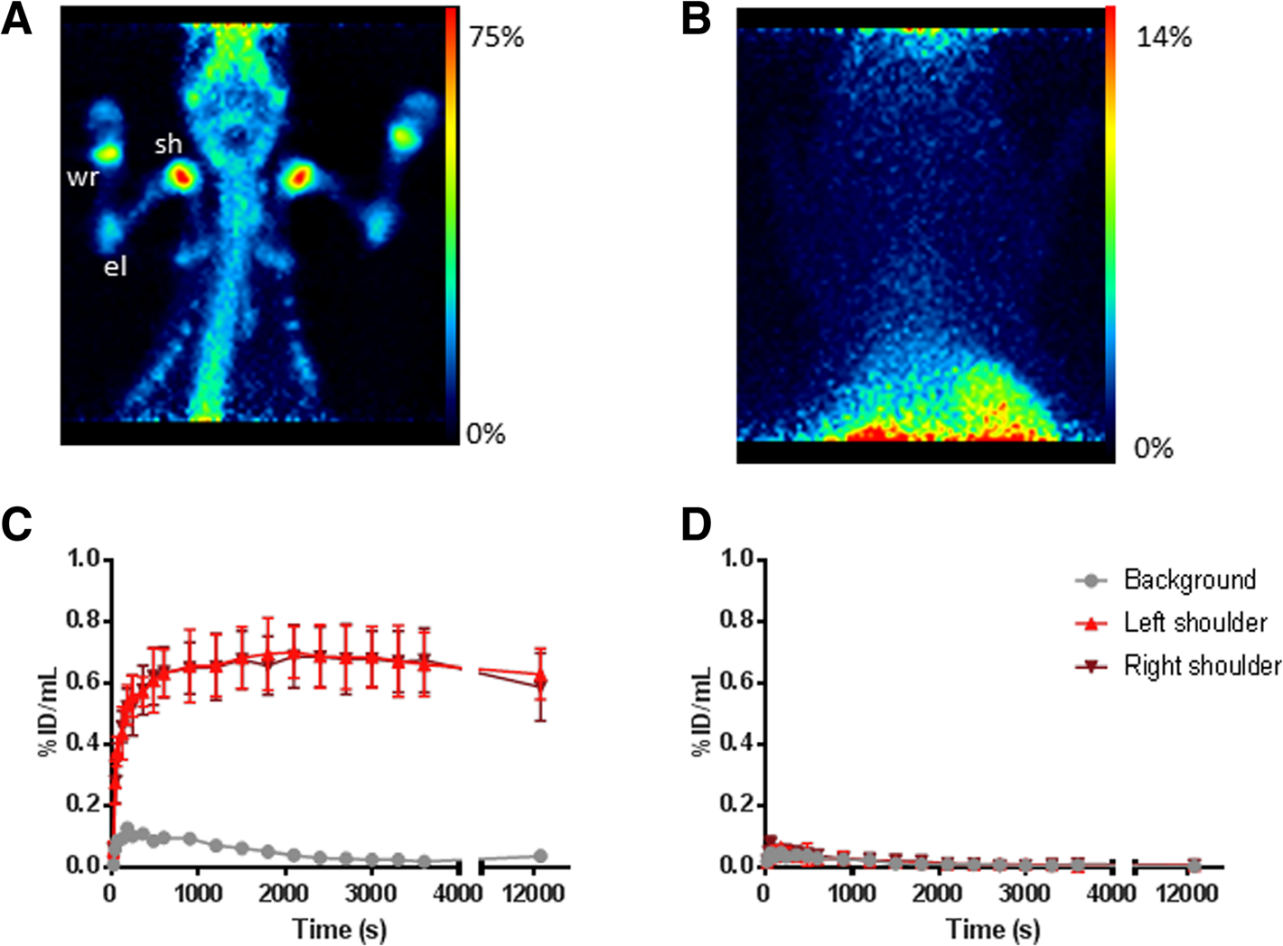
PET analysis of [^44^Sc]**3** in Wistar rats. **a** Maximum intensity projection (MIP) of rat administered with 2 1 h prior to administration of 12 MBq of [^44^Sc]**3**, 4 h p.i of radioactivity; sh shoulder, el elbow, wr wrist. **b** MIP of rat administered with saline 1 h prior to administration of 12 MBq of [^44^Sc]**3**, 4 h p.i. of radioactivity. **c** TAC of radioactivity uptake in the muscle (background grey circles), left shoulder (red triangles), and right shoulder (burgundy triangles) of rat pre-treated with **2** 1 h prior to administering [^44^Sc]**3**. **d** TAC of radioactivity uptake in the muscle (background grey circles) and left shoulder (red triangles) and right shoulder (burgundy triangles) of rat pre-treated with saline 1 h prior to administering [^44^Sc]**3**. Three different ROIs were drawn for each shoulder to calculate the uptake value for the region

Preclinical PET imaging of [^44^Sc]**3** in healthy Wistar rats pre-treated with **2** or saline is summarized in Fig. 2. The total in vivo TCO:Tz ratio was 118:1 based on the amounts of **2** and [^44^Sc]**3** + **1** administered (Additional file 1: Table S1). Small animal PET revealed radioactivity uptake in the shoulders, elbows, wrists, and spinal cord when **2** was administered 1 h prior to [^44^Sc]**3** (Fig. 2a). This uptake was not observed when **2** was not administered (Fig. 2b). Accumulation of [^44^Sc]**3** in the shoulders occurred rapidly and was maintained throughout the duration of image acquisition (60 min) (Fig. 2c). Radioactivity in these areas did not rise above background when **2** was not administered.

### Ex vivo biodistribution

**Fig. 3:**
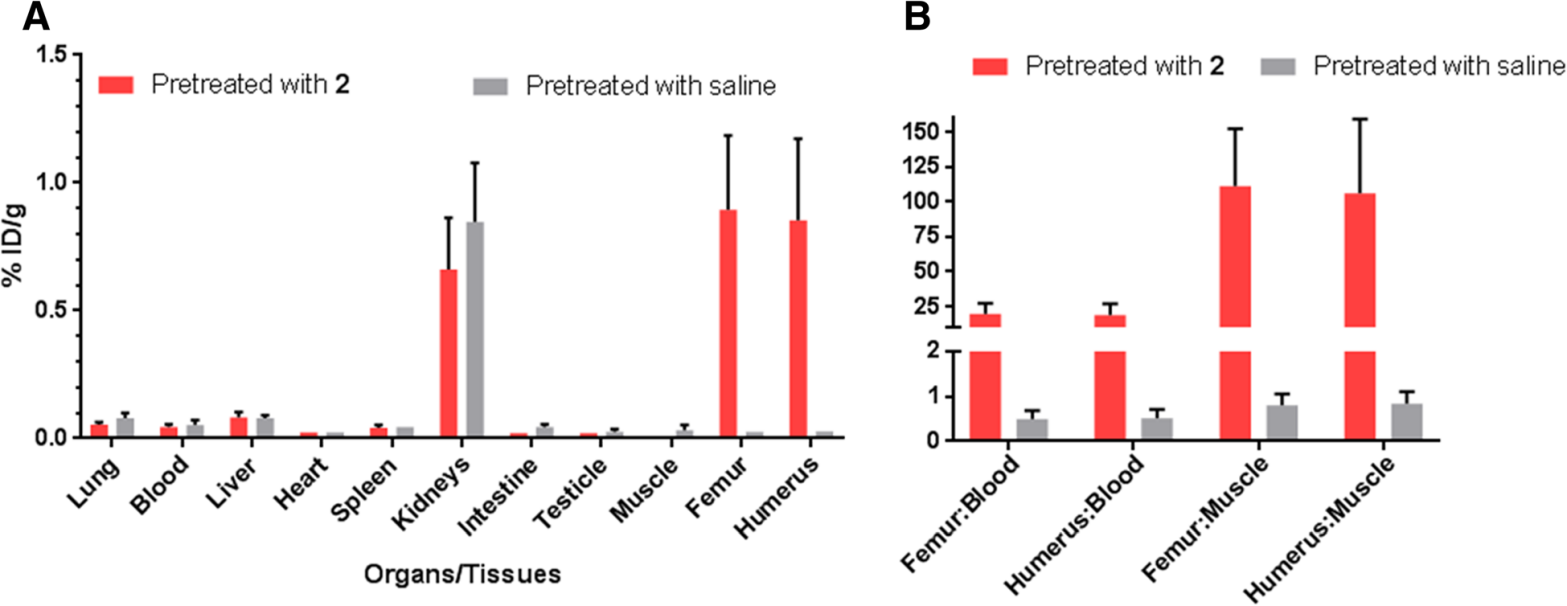
Uptake values of [^44^Sc]**3** in Wistar rats. **a** Ex vivo biodistribution 4 h p.i. of rats pre-treated with **2** or saline 1 h prior to administering 9–12 MBq of [^44^Sc]**3**. **b** Select bone soft tissue ratio of [^44^Sc]**3** in rats pre-treated with 1 or saline

The bone uptake was confirmed with an ex vivo biodistribution study performed 4 h p.i. (Fig. 3). For the rats pre-treated with **2**, the total ratio of TCO:Tz administered was between 114:1 and 134:1 (Additional file 1: Table S1). The uptake in the femur and humerus was 0.9 ± 0.3%ID/g in both areas. Radioactivity in the kidneys was 0.7 ± 0.2% ID/g, while in all other organs and tissues it was below 0.1% ID/g (Fig. 3a). The clearance of [^44^Sc]**3** from non-targeted tissues resulted in high bone-to-soft tissue ratios (Fig. 3b). High bone-to-blood (19.8 ± 0.4 femur, 18.9 ± 0.4 humerus) and bone-to-muscle (111.3 ± 0.4 femur, 106.0 ± 0.5 humerus) ratios were observed (Fig. 3b). When **2** was not administered, the only organs displaying substantial radioactivity were the kidneys (0.8 ± 0.2%ID/g) (Fig. 3a).

## Discussion

A DOTA-functionalized tetrazine (**1**) has been successfully labelled with the PET radionuclide, scandium-44, in high RCY (85–95%) and RCP (> 99%) to produce [^44^Sc]**3**. The Sc^3+^ ion is located within the DOTA cavity in a highly unsymmetric manner. The coordination environment around the Sc^3+^ ion can be both square-antiprismatic and twisted anti-prismic, thus resulting in isomers [32]. In the radiochromatogram for [^44^Sc]**3**, two peaks are present, representing possible isomers formed in solution (Additional file 1: Figure S1). Radio-TLC analysis revealed a change in *R*_f_ value for all radioactive species when **2** was added (Additional file 1: Figure S2). This indicates that all radioactive species were reactive towards the TCO, suggesting that the radioactive peaks in the chromatogram represented isomeric tetrazine species. In a proof-of-concept study, [^44^Sc]**3** was evaluated using dynamic PET imaging concurrently to the ex vivo biodistribution. This allowed real-time monitoring of the shoulder accumulation and clearance from the background tissues. The TAC (Fig. 2c) revealed that the radioactivity accumulation in the shoulder occurred early (ca. 7–9 min) and remained fairly constant over 3.5 h. This gives an indication of the speed of the IEDDA reaction as well as the radiochemical and chemical stability of the dihydropyrole product ([^44^Sc]**4**). Although **2** is a small molecule and should exhibit fast clearance, alendronate has been reported to bind to rat serum proteins [33, 34]. This leaves a slight chance that residual **2** may remain in the blood circulation and be available for an in vivo reaction with [^44^Sc]**3**. Since bisphosphonates are internalized by osteoclasts overtime, a longer pretargeting interval would not be ideal when [^44^Sc]**3** is used as the secondary agent, since it does not penetrate cell membranes. For instance, pretargeted studies using **1** in mice have shown a reduction in bone uptake when a longer pretargeting interval (> 12 h) was used [24]. The half-life of ^44^Sc was beneficial so that any [^44^Sc]**4** formed from the residual **2** would clear from the background tissues (blood and muscle) prior to image acquisition. (Fig. 3b).

The utility of **2** in pretargeted studies has been previously studied as a primary agent to evaluate novel radiolabelled tetrazines for in vivo IEDDA reactions [21, 22, 35]. It is an attractive system in that it does not require a disease model. This is advantageous for evaluating novel tetrazines in higher order species where it would be impossible or unethical to induce a diseased state. In the initial studies, mice were pre-treated with relativity high doses of **2** (20 mg/kg) prior to administering the radiolabelled tetrazine [21, 22, 35]. Although high bone uptake was observed (5–20% ID/g), high radioactivity in the kidneys was also observed (8–13% ID/g). Interestingly, it has been reported that the amount of renal excretion for alendronate increases with concentration in a dose-dependent manner [36]. Although **2** is a modified variant of alendronic acid, all bisphosphonates have the potential to cause renal failure at high concentrations [37]. For example, the bisphosphonates ibandronate and zoledronate can result in renal decline in Wistar rats when doses greater than 1 mg/kg or 3 mg/kg (respectively) are used [37]. Therefore, we opted to decrease the dose of **2** to 3 mg/kg for the current study.

An additional advantage of **2** is that the amount of TCO administered can be easily modified by changing the dose. It has been shown that the number of pretargeted TCO moieties can influence the uptake of the secondary agent in pretargeted studies [35–37]. This is an important factor to consider for clinical translation of the IEDDA reaction as increasing the primary agent can lead to toxicity. We have shown that by lowering the dose of **2**, yet maintaining a high ratio of the TCO:Tz administered, pretargeted imaging was possible in a larger rodent model.

## Conclusions

Radiolabelling of a DOTA-functionalized tetrazine with generator-produced scandium-44 was optimized and implemented. The labelled tracer ([^44^Sc]**3**) exhibited high in vitro stability and was subsequently used for pretargeted imaging in rats via the IEDDA reaction. A TCO-modified bisphosphonate, alendronate, was used as the primary agent, and bone imaging was successful using [^44^Sc]**3**. The half-life of scandium-44 was suitable to obtain good clearance from non-targeted tissues giving rise to high T:NT ratios and excellent image quality. Thus far, all of the tetrazines radiolabelled with typical therapeutic nuclides (i.e., *α* or *β*^−^ emitters) found in the literature contain a DOTA-metal complex (for a review, see [6]). Given the stability of Sc-DOTA complexes, the convenient ^44^Sc-half-life, and the high-quality ^44^Sc-PET images, scandium-44 is an attractive radionuclide to develop companion diagnostics for these agents. Furthermore, we were able to demonstrate that pretargeted imaging via IEDDA can be performed in rodents larger than the typical mouse models previously used.

## Additional files

Additional file 1: **Figure S1.** HPLC radiochromatogram chromatogram of [^44^Sc]**3** (*R*_*t*_ = 5.7 min). **Figure S2.** In vitro stability of [^44^Sc]**3**. (A) radio-TLC analysis of [^44^Sc]**3** with 2 (lane 2) and without 2 (lane 1) following radiosynthesis. (B) Percent intact over time after incubation in saline (red circles) and human serum albumin (blue squares) for 0.5–24 h at 37 °C. **Table S1.** Summary of the uptake for [^44^Sc]**3** in Wistar rats. **Table S2.** Summary of bone uptake values (4 h p.i.) and TCO:Tz ratios in individual rats. (DOC 275 kb)

## Abbreviations

*A*_m_: Molar activity
DOTA: 1,4,7,10-Tetraazacyclododecane-1,4,7,10-tetraacetic acid
EOS: End of synthesis
IEDDA: Inverse electron demand Diels-Alder
MIP: Maximum intensity projection
ndc: Non-decay corrected
PET: Positron emission tomography
RCY: Radiochemical yield
RT: Room temperature
TAC: Time-activity curve
TCO: *Trans*-cyclooctene
Tz: Tetrazine
