# Supplementary material for: Evaluation of the inverse electron demand Diels-Alder reaction in rats using a scandium-44-labelled tetrazine for pretargeted PET imaging
Source: EJNMMI Res. 2019 May 28;9:49. doi: 10.1186/s13550-019-0520-y (PMC6538705; doi:10.1186/s13550-019-0520-y)
Supplement: Supplementary file 1 — Figure S1. HPLC radiochromatogram chromatogram of [44Sc]3 (Rt = 5.7 min). Figure S2. In vitro stability of [44Sc]3. (A) radio-TLC analysis of [44Sc]3 with 2 (lane 2) and without 2 (lane 1) following radiosynthesis. (B) Percent intact over time after incubation in saline (red circles) and human serum albumin (blue squares) for 0.5–24 h at 37 °C. Table S1. Summary of the uptake for [44Sc]3 in Wistar rats. Table S2. Summary of bone uptake values (4 h p.i.) and TCO:Tz ratios in individual rats. (DOC 275 kb) [file 13550_2019_520_MOESM1_ESM.doc]

**SUPPORTING INFORMATION**

**Evaluation of the inverse electron demand Diels Alder reaction in rats using a scandium-44 labelled tetrazine for pretargeted PET imaging**

Patricia E. Edem^1,2,3^, Jean-Philippe Sinnes^4^, Stefanie Pektor^5^, Nicole Bausbacher^5^, Raffaella Rossin^7^, Abdolreza Yazdani^6,8^, Matthias Miederer^5^, Andreas Kjær^1,2^, John F. Valliant^6^, Marc S. Robillard^7^, Frank Rösch^4^, Matthias M. Herth^1,3*^

^1^Department of Clinical Physiology, Nuclear Medicine & PET, Rigshospitalet, Blegdamsvej 9, 2100 Copenhagen, Denmark. ^2^Cluster for Molecular Imaging, Department of Biomedical Sciences, University of Copenhagen, Blegdamsvej 3, 2200 Copenhagen Denmark. ^3^Department of Drug Design and Pharmacology, University of Copenhagen, Jagtvej 162, 2100 Copenhagen, Denmark. ^4^Johannes Gutenberg-Universität Mainz, Saarstraße 21, 55122, Mainz, Germany. ^5^University Medical Center Mainz, Langenbeckstr. 1, 55131 Mainz, Germany. ^6^McMaster University, 1280 Main St. W, L8S 4L8, Hamilton, ON, Canada. ^7^Tagworks Pharmaceuticals, Geert Grooteplein Zuid 10, 6525 GA Nijmegen, The Netherlands. ^8^Pharmaceutical Chemistry and Radiopharmacy Department, School of Pharmacy, Shahid Beheshti University of Medical Sciences, P O Box: 14155-6153, Tehran, Iran.

Contents

[1. HPLC Chromatograms for [^44^Sc]3 2](#__RefHeading___Toc6420186)

[2. In vitro stability for [^44^Sc]3 2](#__RefHeading___Toc6420187)

[3. Summary of bone uptake for [^44^Sc]3 3](#__RefHeading___Toc6420188)

# 1. HPLC Chromatogram for [^44^Sc]3

**
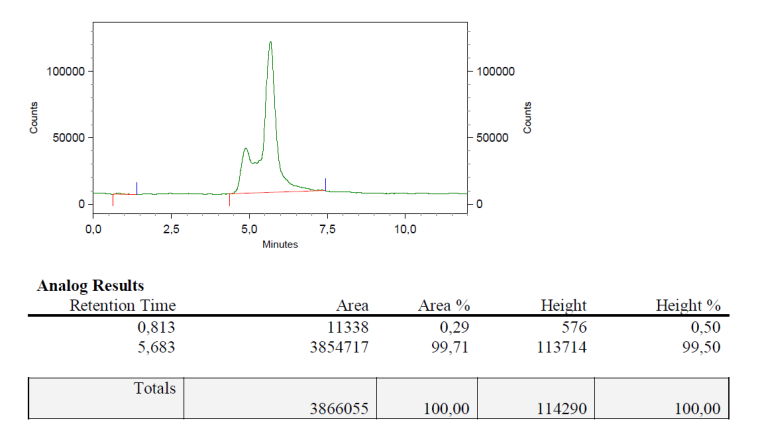
**

**Figure S 1**. HPLC radiochromatogram chromatogram of [^44^Sc]**3** (R_t_ = 5.7 min).

# 2. In vitro stability for [^44^Sc]3

**Figure S 2**. In vitro stability of [^44^Sc]**3**. (A) radio-TLC analysis of [^44^Sc]**3** with **2** (lane 2) and without **2** (lane 1) following radiosynthesis. (B) Percent intact over time after incubation in saline (red circles) and human serum albumin (blue squares) for 0.5-24 h at 37 °C.


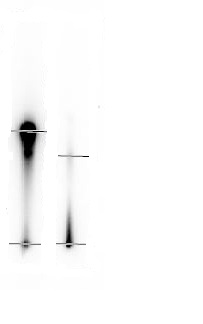


[^44^Sc]**3**

[^44^Sc]**4**

A)

B)

# 3. In vivo Evaluation of [^44^Sc]3

**Table 1**. Summary of the uptake for [^44^Sc]**3** in Wistar rats

|  | **Pretreated with 2** | **Pretreated with saline** |
| --- | --- | --- |
| **Tissue/Organ** | **%ID/g** | **%ID/g** |
| **Lung** | 0.05 ± 0.01 | 0.08 ± 0.02 |
| **Blood** | 0.04 ± 0.01 | 0.05 ± 0.02 |
| **Liver** | 0.08 ± 0.02 | 0.08 ± 0.01 |
| **Heart** | 0.02 ± 0.00 | 0.02 ± 0.00 |
| **Spleen** | 0.04 ± 0.01 | 0.05 ± 0.00 |
| **Kidneys** | 0.7 ± 0.2 | 0.8 ± 0.2 |
| **Intestine** | 0.02 ± 0.00 | 0.05 ± 0.01 |
| **Testicle** | 0.02 ± 0.00 | 0.03 ± 0.01 |
| **Muscle** | 0.008 ± 0.000 | 0.03 ± 0.02 |
| **Femur** | 0.9 ± 0.3 | 0.03 ± 0.00 |
| **Humerus** | 0.9 ± 0.3 | 0.03 ± 0.00 |

**Table 2**. Summary of bone uptake values (4 h p.i.) and TCO:Tz ratios in individual rats

| **Entry** | **TCO:Tz Ratio Administered** | **% ID/g Femur** | **% ID/g Humerus** |
| --- | --- | --- | --- |
| **1** | 115:1 | 0.62 | 0.44 |
| **2** | 116:1 | 1.17 | 0.96 |
| **3** | 117:1 | 1.13 | 1.36 |
| **4** | 134:1 | 0.66 | 0.64 |
